# Extracellular vesicles from human embryonic stem cell-derived cardiovascular progenitor cells promote cardiac infarct healing through reducing cardiomyocyte death and promoting angiogenesis

**DOI:** 10.1038/s41419-020-2508-y

**Published:** 2020-05-11

**Authors:** Qiang Wu, Jinxi Wang, Wilson Lek Wen Tan, Yun Jiang, Shihui Wang, Qiang Li, Xiujian Yu, Jiliang Tan, Shenyan Liu, Peng Zhang, Zenia Tiang, Zhongyan Chen, Roger Sik-Yin Foo, Huang-Tian Yang

**Affiliations:** 1 CAS Key Laboratory of Tissue Microenvironment and Tumor, Laboratory of Molecular Cardiology, Shanghai Institute of Nutrition and Health, University of Chinese Academy of Sciences (CAS), CAS, Shanghai, 200031 P. R. China; 2 Human Genetics, Genome Institute of Singapore, Singapore, 138672 Singapore; 3 Cardiovascular Research Institute, Yong Loo Lin School of Medicine, National University of Singapore, Singapore, 117599 Singapore; 4 Institute for Stem Cell and Regeneration, CAS, Beijing, 100101 P. R. China

**Keywords:** Apoptosis, Embryonic stem cells

## Abstract

Human pluripotent stem cells (hPSCs)-derived cardiovascular progenitor cells (CVPCs) are a promising source for myocardial repair, while the mechanisms remain largely unknown. Extracellular vesicles (EVs) are known to mediate cell–cell communication, however, the efficacy and mechanisms of hPSC-CVPC-secreted EVs (hCVPC-EVs) in the infarct healing when given at the acute phase of myocardial infarction (MI) are unknown. Here, we report the cardioprotective effects of the EVs secreted from hESC-CVPCs under normoxic (EV-N) and hypoxic (EV-H) conditions in the infarcted heart and the long noncoding RNA (lncRNA)-related mechanisms. The hCVPC-EVs were confirmed by electron microscopy, nanoparticle tracking, and immunoblotting analysis. Injection of hCVPC-EVs into acutely infracted murine myocardium significantly improved cardiac function and reduced fibrosis at day 28 post MI, accompanied with the improved vascularization and cardiomyocyte survival at border zones. Consistently, hCVPC-EVs enhanced the tube formation and migration of human umbilical vein endothelial cells (HUVECs), improved the cell viability, and attenuated the lactate dehydrogenase release of neonatal rat cardiomyocytes (NRCMs) with oxygen glucose deprivation (OGD) injury. Moreover, the improvement of the EV-H in cardiomyocyte survival and tube formation of HUVECs was significantly better than these in the EV-N. RNA-seq analysis revealed a high abundance of the lncRNA MALAT1 in the EV-H. Its abundance was upregulated in the infarcted myocardium and cardiomyocytes treated with hCVPC-EVs. Overexpression of human MALAT1 improved the cell viability of NRCM with OGD injury, while knockdown of MALAT1 inhibited the hCVPC-EV-promoted tube formation of HUVECs. Furthermore, luciferase activity assay, RNA pull-down, and manipulation of miR-497 levels showed that MALAT1 improved NRCMs survival and HUVEC tube formation through targeting miR-497. These results reveal that hCVPC-EVs promote the infarct healing through improvement of cardiomyocyte survival and angiogenesis. The cardioprotective effects of hCVPC-EVs can be enhanced by hypoxia-conditioning of hCVPCs and are partially contributed by MALAT1 via targeting the miRNA.

## Introduction

Myocardial infarction (MI), characterized by massive cardiomyocyte death followed by cardiac dysfunction and myocardial fibrosis, is a leading cause of death worldwide^1–3^. Endogenous myocardial protective systems can be triggered by injurious stimuli to reduce cardiomyocyte death and promote proliferation of preexisting cardiomyocytes but they are insufficient for cardiac repair^4–10^. Therefore, developing new therapeutic strategies to promote the infarct healing by reducing cardiomyocyte death following acute MI (AMI) and improving the cardiac performance are desirable^11^.

Cell therapy by transplantation of stem/progenitor cells and their derived cardiovascular cells is one of the most notable alternative therapeutic approaches under exploration^12–18^. Among them, human pluripotent stem cells (hPSCs), including both human embryonic stem cells (hESCs) and human induced pluripotent stem cells (hiPSCs), hold promise for promoting cardiac infarct healing because they can be theoretically produced in unlimited quantities of cells of any lineages, including cardiomyocytes^17,19–24^. hESC-derived cardiovascular progenitor cells (hCVPCs) have been shown to improve cardiac function of rodent infarcted hearts when implanted during the subacute stage of ischemia/reperfusion (I/R) hearts^14^. We found that SSEA1^+^ hCVPCs promote cardiac infarct healing when given during the early phase of MI in mouse^25^ and nonhuman primate^26^ models, showing the significant improvement in the recovery of left ventricular (LV) function and the amelioration of cardiomyocyte death. Moreover, the adverse effects of proarrhythmia and tumorigenicity are not observed in the patients with advanced ischemic heart failure (HF) after transplanted SSEA1^+^ hCVPCs^27,28^. More recently, we found that the beneficial effects of these cells to the infarcted hearts are related to the hCVPC-secreted cytokines via the modulation of macrophage polarization towards a reparative phenotype in the post-MI hearts^25^, while the secretome of hCVPCs and their functions in the cardiac protection remain largely unknown.

The cumulative evidence has revealed that extracellular vesicles (EVs)/exosomes contribute critically to the cardioprotective effects of transplanted stem cells and their derivatives^9,29–31^. It has been shown that administration of the EVs either secreted from hESC- or hiPSC-derived SSEA1^+^-CVPCs (hCVPC-EVs) at 3 weeks post MI improves recovery of cardiac function in the mouse model^32^, in part, through the specific microRNA (miRNA) signature contained in the EVs^33,34^. EVs contain proteins, lipids, and nucleic acids (mRNAs, miRNAs, etc.) with important functions^35^. However, it is unclear whether the hCVPC-secreted EVs (hCVPC-EVs) have benefits for promoting cardiac infarct healing when given at the acute phase of MI and, if yes, how they work.

The miRNAs in EVs derived from various types of cells, such as mesenchymal stem cells (MSCs)^36,37^, Sca1^+^ adult cells^38^, cardiosphere-derived cells^39^, and mouse ESCs^40^, have been shown to protect the heart from MI injury. However, whether the other type of RNAs, such as long noncoding RNAs (lncRNAs), contain in the EVs and what functions they have need more studies, though recent studies showed that the lncRNAs are detected in the EVs derived from human ESC/iPSC-derived cardiomyocytes^41^ and LncRNA H19 mediates, at least partially, the cardioprotective role of EVs derived from MSCs in promoting angiogenesis^42^. It has been shown that a lncRNA metastasis-associated lung adenocarcinoma transcript 1 (MALAT1) contributes to vascular growth in a mouse retinal angiogenesis model^43^ and human umbilical cord MSC-derived EVs prevent aging-induced cardiac dysfunction by releasing lncRNA MALAT1^44^. However, it is unknown whether hCVPC-EVs contain lncRNAs and whether MALAT1 contributes to the cardiac protection of hCVPC-EVs through targeting miRNAs.

In the present study, using a murine permanent MI model involving the administration of SSEA1^+^-hCVPC-secreted EVs during AMI, combining in vivo and in vitro analysis, we investigated (i) whether hCVPC-EVs improve infarct healing; (ii) whether hCVPC-EVs contain lncRNA candidates benefiting cardiac protection; and (iii) what the miRNA target of hCVPC-EV-contained lncRNA MALAT1 confers the protection of MALAT1 in cardiomyocyte survival and angiogenesis. Our findings have identified previously unrecognized function of hCVPC-EVs in promoting infarct healing when given at the acute phase of MI and provided new insights into the mechanisms underlying the beneficial effects of hCVPC-EVs in cardiac protection.

## Materials and methods

### hESC culture, hCVPC induction, and hypoxia treatment

hPSCs (hESC line H9, WiCell) were routinely maintained in mTeSR1 media (Stem Cell Technologies) on Matrigel-coated plates (hESC qualified, Corning) according to manufacturer’s instructions as described previously^21,25,26,45^. The CVPC induction was performed as described previously^21,25,26,45^. Briefly, undifferentiated hESCs were cultured until 80–90% confluent, dissociated with Accutase (Stem Cell Technologies), and then plated onto Matrigel-coated culture dishes at a density of 5 × 10^4^ cells/cm^2^ in the CVPC-induction medium (DMEM/F12 (Gibco), 1× B27 supplement without vitamin A (Gibco), 1% l-Glutamine (Gibco), 1% penicillin/streptomycin (Gibco), 400 µM 1-thioglycerol (Sigma), 50 µg/mL ascorbic acid (Sigma), 25 ng/mL bone morphogenetic protein 4, and 3 µM glycogen synthase kinase 3 inhibitor (CHIR99021, Stemgent)). To enhance cell viability, a Rho-associated protein kinase inhibitor (Y27632, 5 µM; Calbiochem) was added on the first day of differentiation and removed via medium change 24 h later. hPSC-CVPCs were harvested for cell transplantation and the cultured medium was collected for EV isolation after 3 days of differentiation.

Hypoxic conditioning was performed by placing the cells in an oxygen control cabinet (Ruskinn, England) mounted within an incubator and equipped with oxygen controller and sensor for continuous oxygen level monitoring for 48 h in DMEM basal medium. The oxygen concentration in the cabinet was maintained at 1%, with a residual gas mixture composed of 5% CO_2_ and balanced N_2_. Normoxic conditioning hCVPCs were incubated under 21% O_2_ and 5% CO_2_ for 48 h in DMEM basal medium. Both normoxic and hypoxic conditioned medium were collected for EV isolation.

### Flow cytometry analysis

Flow cytometry was performed as described previously^21^. Cells to be examined were harvested, dissociated with Accutase (Stem Cell Technologies). The samples were stained for the presence of cardiac progenitor marker PE-conjugated SSEA1 (1:20; eBioscience), and then analyzed and quantified by flow cytometry (FACStar Plus Flow Cytometer, BD bioscience).

### Immunocytochemical staining analysis

The cells were immunostained as described previously^21^. Briefly, cells were fixed with 4% paraformaldehyde, permeabilized in 0.3% Triton X-100 (Sigma), blocked in 10% normal goat serum (Vector Laboratories), and then incubated at 4 °C overnight with primary antibodies against MESP1 (1:100, Aviva Systems Biology), MEF2C (1:100, Cell Signaling), GATA4 (1:300, Santa Cruz Biotechnology), ISL1 (1:100, Developmental Studies Hybridoma Bank), and NKX2-5 (1:200, Santa Cruz Biotechnology). Primary antibodies were detected with DyLight 549-conjugated secondary antibodies, and nuclei were counterstained with DAPI (Sigma).The immunostaining of isolated NRCMs were stained with α-actinin antibody and terminal deoxynucleotidyl transferase-mediated dUTP nick end labeling (TUNEL) using the In situ Cell Death Detection Kit (Roche Applied Science, Germany) to detect the cell death.

### EV isolation

The EV-N and EV-H were isolated from normoxic and hypoxic conditioned medium separately as described previously^46^. Briefly, The collected hCVPC conditional medium was centrifugated at 300×*g* for 30 min followed by 2000×*g* for 30 min, 4 °C to remove cells and dead cells, and then centrifugated at 10,000×*g* for 30 min, 4 °C to remove cell debris, finally centrifugated twice at 100,000×*g* for 70 min, 4 °C with a SW-41 rotor (Beckman Coulter), followed by washing with phosphate-buffered saline (PBS). The final pellet containing EVs was resuspended in PBS and analyzed by NanoSight NS300 (Malvern Panalytical), transmission electron microscope and Western blot, or lysed with QIAzol reagent (#217084, Qiagen) for RNA analysis.

### Nanoparticle tracking analysis (NTA)

The NTA was carried out to determine the EV size and concentration by using NanoSight NS300 (Malvern Panalytical) on the isolated EVs as previously reported^38^. The isolated EV pellet as described in the above EV Isolation method was resuspended in PBS, and then 10 μL of it was used for NTA (the sample was diluted to 700 μL with PBS), and 10 μL of it was used for Pierce BCA Protein Assay. During NTA analysis, three 30 s video taken per sample were averaged as one value and five samples were examined in each group. The PBS was subtracted from particle number/mL after quantification. The analysis was performed by using the NTA software (NTA 3.2 Dev Build 3.2.16). Based on the measurement from NTA and Pierce BCA Protein Assay, the 1 μg EV protein had 32.80 ± 8.529 × 10^8^ of particles in the EVs secreted from hESC-CVPCs under normoxic cultivation (EV-N) group and 34.60 ± 11.76 × 10^8^ of particles in the EVs secreted from hESC-CVPCs under hypoxic cultivation (EV-H) group as shown in Supplementary Fig. S1. Accordingly, the 20 μg EV protein contained about 485–827 × 10^8^ particles in the EV-N group, and about 457–927 × 10^8^ particles in the EV-H group (*p* > 0.05, *n* = 5).

### Western blot analysis

The cells and the EVs were lysed with the radioimmunoprecipitation assay lysis buffer. Then, the protein extracts were separated via gel electrophoresis, transferred to a polyvinylidene fluoride membrane, and blocked with 5% bovine serum albumin for 1 h. The membranes were incubated overnight with primary antibody against Alix (1:1000, Cell Signaling Technology), TSG101 (1:1000, Abcam), CD63 (1:200, Santa Cruz Biotechnology), CD81 (1:500, Thermo Fisher Scientific), and CD9 (1:500, Thermo Fisher Scientific). Then, the membranes were incubated with horseradish peroxidase-conjugated secondary antibodies at room temperature for 2 h and exposed via enhanced chemiluminescence.

### MI and EV treatment

All surgical procedures described were performed in accordance with the Guidelines for Care and Use of Laboratory Animals published by the US National Institutes of Health (NIH Publication, 8th Edition, 2011) and were approved by the Institutional Animal Care and Use Committee of Shanghai Institutes for Biological Sciences. The male C57BL/6 mice aged 10–12 weeks were randomly divided into four groups: Sham ^+^ PBS (*n* = 8), MI + PBS (*n* = 12), MI + EV-N (*n* = 12), and MI + EV-H (*n* = 15) after excluded the LV ejection fraction (LVEF) values greater than 50% (2 in MI + PBS, and 1 in MI + EV-H) at day 2 post MI. The mice were anesthetized via intraperitoneal injection of 50 mg/kg sodium pentobarbital, ventilated with a volume-regulated respirator (SAR830, Cwe Incorporated), and the MI model was induced through the permanent ligation of the left anterior descending (LAD) coronary artery with a 10-0 Prolene suture as previously described^25,40,47^. Of 20 μg EVs, mixed with PBS in a total volume of 20 μL, were directly injected into two sites in the border zones of infarcted myocardium (10 μL each site) after the ligation of LAD coronary artery, and the equal amount of PBS was injected into the sham heart too. Then the muscle and skin of the chest were then sutured with 6-0 and 5-0 Prolene suture, respectively. The body temperature was maintained at 37 °C during the surgical procedure.

### Echocardiography

Transthoracic echocardiography (Vevo 2100, Visual Sonics) with a 25-MHz imaging transducer was performed on isoflurane anesthetized mice to measure the heart function as previously described^25^. LVEF and LV systolic dimensions (LVDs) were recorded and the average values were collected from each of three consecutive cardiac cycles.

### Immunohistochemical staining

Heart tissues were embedded in OCT (SAKURA) compound for histological analysis. were prepared at 400 μm intervals. For fluorescent immunohistochemistry, Transversal frozen sections (5 μm) were fixed with 4% paraformaldehyde, permeabilized in 0.4% Triton X-100 (Sigma), and stained with anti-CD31, α-SMA, and cTnT antibody which were detected by fluorescent conjugated secondary antibodies. Nuclei were stained with DAPI (Sigma). Cell death was evaluated with an In situ Cell Death Detection Kit (Roche Applied Science, Germany) for TUNEL staining as directed by the manufacturer’s instructions. The percentage of TUNEL^+^ cardiomyocytes was quantified as the ratio of TUNEL^+^ cTnT^+^ to total cTnT^+^ cells. The immunostaining images were blindly captured using a Zeiss inverted microscope and processed using ZEN software. Three microscopic fields were quantified for each slice. The Image-processing software (Image J) was used as the image quantification software.

### Masson’s trichrome staining

For the Masson’s trichrome-stained images, each heart was made five slices from the point of ligation to the apex of the heart. The morphometric parameters in the five slices of each heart including total LV area and scar area were blindly analyzed, and the scar size was calculated as the total scar area divided by the LV area as described previously^25,47^.

### RNA extraction

Total RNA was isolated from hCVPC-EVs using miRNeasy Micro Kit (Qiagen) according to the manufacturer’s instructions. The concentration of RNA fraction was quantified using Nanodrop (Thermo Fisher Scientific) according to the manufacturer’s protocol.

### RNA-sequencing (RNA-seq) analysis

Five EV samples from each of EV-N and EV-H groups from H9 hESC origin were proceeded for RNA-seq analysis as previously reported^48,49^. Total RNA sequencing was performed with the Truseq Stranded Total RNA Library Prep kit (Illumina, RS-122-2201). This made use of Ribo-Zero to remove abundant cytoplasmic rRNA from total RNA samples. Remaining intact RNA was fragmented using a chemical mix, followed by first- and second-strand cDNA synthesis using random hexamer primers. “End-repaired” fragments are ligated with unique Illumina proprietary adapters. All individually indexed samples were pooled together and multiplexed for sequencing. Libraries were sequenced using the Illumina Hiseq 2500 sequencing system and paired-end 101 bp reads were generated for analysis. Fastq files were aligned against human reference (hg19/hGRC37) using the Tophat2. Duplicate reads were removed using MarkDuplicates from Picard tools, and per gene read counts for Ensembl (v75) gene annotations were computed using htseq-count. Differential gene expression analysis was performed using R-package EdgeR. Principal component analysis was computed using R function prcomp. Hierarchical clustering and heatmaps were generated using R.

### Target prediction of MALAT1

The miRNA targets of MALAT1 were predicted by miRcode (http://mircode.org/). The binding of MALAT1 and miRNAs was also predicted by DianaTools (http://carolina.imis.athena-innovation.gr/diana_tools/web/index.php?r=lncbasev2%2Findex-predicted).

### Quantitative reverse transcription polymerase chain reaction (RT-qPCR)

For miRNAs, total RNA was reverse-transcribed into cDNA by the miRcute Plus miRNA First-Strand cDNA Synthesis Kit (#KR211-02, Tiangen Biotech) and subsequently determined using a miRcute Plus miRNA qPCR Detection Kit (SYBR Green) (#FP411-02, Tiangen Biotech) with Qiagen predesigned primers. All kits were used according to the manufacturer’s instructions. A U6 transcript was used as an internal control to normalize RNA input. For lncRNA, total RNA was reverse-transcribed into cDNA by the ReverTra Ace^®^ (#TRT-101, TOYOBO) with random primers and subsequently determined using a FastStart Universal SYBR Green Master Kit (#4913914001, Roche) with specific lncRNA primers. All kits were used according to the manufacturer’s instructions. lncRNA levels were normalized to glyceraldehyde 3-phosphate dehydrogenase (GAPDH). Human MALAT1 (hMALAT1) primer: hMALAT1 (forward): 5′-ctaggactgaggagcaagcg-3′; hMALAT1 (reverse): 5′-accaaatcgttagcgctcct-3′.

### Isolation, culture, and oxygen glucose deprivation (OGD) treatment of cardiomyocytes

Isolation of neonatal rat cardiomyocytes (NRCMs) were performed as previously reported^50^. Briefly, the NRCMs were prepared by enzymatic digestion of hearts obtained from newborn (1 day old) Sprague–Dawley rat pups and plated on cell culture grade plates (coated with gelatin) at a density of 5 × 10^5^ cells/cm^2^ in DMEM/F12 medium and maintained at 37 °C in humid air with 5% CO_2_. NRCMs were subjected to hypoxia in vitro in an oxygen control cabinet (Ruskinn, England) mounted within an incubator and equipped with oxygen controller and sensor for continuous oxygen level monitoring. A mixture of 85% nitrogen, 10% hydrogen, and 5% CO_2_ was utilized to create hypoxia and the O_2_ in the chamber was monitored and maintained at a level <0.1%. The NRCMs were treated with OGD injury by cultured in the culture medium without serum and glucose and in hypoxic condition (<0.1% O_2_) for 6 h as previously reported^51^. The EV-N or EV-H (1 μg/mL), miR-497-mimic (Ribobio, Guangzhou, China) or/and pcDNA3.1(-)-MALAT1 plasmid (Shanghai Integrated Biotech Solutions), MALAT1 GapmeR or control (Guangzhou Epibiotek) with Lipofectamine 3000 (Thermo Fisher Scientific) were added into the culture medium of the NRCMs. Then, the cardiomyocytes were stained by TUNEL, cTnT, and DAPI to evaluate the level of cell death in the NRCMs. The Image-processing software (Image J) was used to quantify the TUNEL^+^ nucleus and total nucleus. The Cell Counting Kit-8 (Sigma-Aldrich) was applied and absorbance of formazan dye produced by living cells was measured in an Infinite M200 Microplate Reader (Tecan, Maennedorf, Switzerland). The culture medium from various groups was collected to measure the activity of lactate dehydrogenase (LDH) release with a LDH Release Assay Kit (Beyotime, China).

Isolation of adult mouse cardiomyocytes (AMCMs) were performed by a langendorff-based approach as previously reported^52^. Briefly, male C57BL/6 mice aged 10–12 weeks were anesthetized with pentobarbital (50 mg/kg body weight, i.p.), then the chest was opened to lift the heart and cut the aorta. The heart was then attached to the perfusion cannula through the aorta and perfused with the digestion buffer (Collagenase II 0.5 mg/mL (Worthington, USA), Protease XIV 0.05 mg/mL (Sigma-Aldrich, Singapore)) for 10–15 min at the flow rate of 4 ml/min following 3 min of perfusion in the perfusion buffer. Once appeared flaccid, the heart was removed from the cannula and the ventricle was cut into sections followed by dissociated the cells with a plastic pipette in digestion buffer. Further, the cell suspension underwent gravity settling for four rounds within Ca^2+^ reintroduction buffers. The cells resuspended with a plating medium were plated onto laminin (5 μg/mL, Thermo Scientific, Singapore) coated culture dish after passed through a 100-μm filter, in a humidified tissue culture incubator (37 °C, 5% CO_2_), and after 1 h, the cells were cultivated in the culture medium^53^. The AMCMs were treated with hCVPC-EVs (1 μg/mL) and PBS (equal volume) for 24 h, and then were washed twice with PBS before RNA extraction and RT-qPCR.

### Migration and tube formation assays

Human umbilical vein endothelial cells (HUVECs) (Shanghai Zhongqiao Xinzhou Biotechnology) were cultured with the endothelial cell medium (ECM) (Sciencell, #1001). The ECM consists of basal culture medium, supplemented with 5% fetal bovine serum (FBS, Sciencell, #0025), 1% endothelial growth factor (ECGS, Sciencell, #1052), and 1% penicillin/streptomycin solution (P/S, Sciencell, #0503). Cell migration assay was performed as previously reported^54,55^, with 10 μg/mL mitomycin (Sigma M0503) treated for 2 h, the 100% confluent monolayers of HUVEC were scratched and washed three times with PBS. Then the hCVPC-secreted EV-N or EV-H (1 μg/mL), or PBS (equal volume to the EVs) were added into the 12-well plates seeded with HUVECs. The marked areas were captured by using a Zeiss inverted microscope before and after different treatments for 24 h^33^.

For tube formation assay, 48-well plates were coated with 200 μL of Growth Factor Reduced Matrigel (Corning) per well and incubated at 37 °C for 30 min. HUVECs were seeded at a density of 5 × 10^4^ cells/cm^2^ in the DMEM Basal Medium and maintained at 37 °C in humid air with 5% CO_2_ for 8 h. The EV-N or EV-H (1 μg/mL), miR-497-mimic (Ribobio, Guangzhou, China) or/and pcDNA3.1(−)-MALAT1 plasmid (Shanghai Integrated Biotech Solutions), MALAT1 GapmeR or control (Guangzhou Epibiotek) with Lipofectamine 3000 (Thermo Fisher Scientific) were added into the culture medium of the HUVECs. Capillary-like structure formation by HUVECs was recorded using a Zeiss inverted microscope. The Image J was used to quantify thetubelength^40^.

### Luciferase activity

To verify the direct interactions between MALAT1 and miR-497, the hMALAT1 cDNA full length (8779 bp) was cloned into psiCHECK2 (Promega, Madison, WI) from the pcDNA3.1(−)-MALAT1 plasmid (Shanghai Integrated Biotech Solutions) between the XhoI and NotI restriction endonuclease sites. The map of psiCHECK2-MALAT1 plasmid was shown in Supplementary Fig. S2. The renilla luciferase, hRluc, was used to monitor changes in expression as the result of MALAT1 and miRNA interaction. Synthetic firefly luciferase gene (hluc+) was the control luciferase. SV40 promoter was the promotor of hRluc and MALAT cDNA full length sequence. Lipofectamine 3000 (Thermo Fisher Scientific) was used to cotransfect the plasmids together with synthetic negative control or miR-150- or miR-497-mimic (Ribobio, Guangzhou, China) into HEK293T cells growing in 48‐well plates. Twenty-four hours later, the cells were lysed in 1× passive lysis buffer, and the activities of Renilla luciferase and firefly luciferase were determined with the Dual Luciferase Reporter Assay System (Promega).

### RNA pull down assay

The biotin labeled has-miR-497-5p (bio-miR497-5p) was purchased from Guangzhou RiboBio. Bio-miR-497-5p (100 nM) was transfected into HUVECs (about 50% confluent) with Lipofectamine 3000 (Thermo). After 24 h of transfection, the cell lysate was collected and pulled down the RNA with Streptavidin Magnetic Beads (Thermo, Cat:88816)^56^, then measured relative abundance of hMALAT1 by RT-qPCR.

### Statistics

Data are expressed as mean ± SEM. Statistical significance was analyzed by using the unpaired Student’s *t* test or one-way analysis of variance (ANOVA) followed with Bonferroni’s multiple as appropriate. Two-way ANOVA was applied with Tukey’s multiple comparison for analysis of echocardiographic data. Statistical analyses were performed with Graphpad Prism software (version 6.1). A *p* value <0.05 was considered statistically significant.

## Results

### Characterization of hCVPC-secreted EVs

**Fig. 1:**
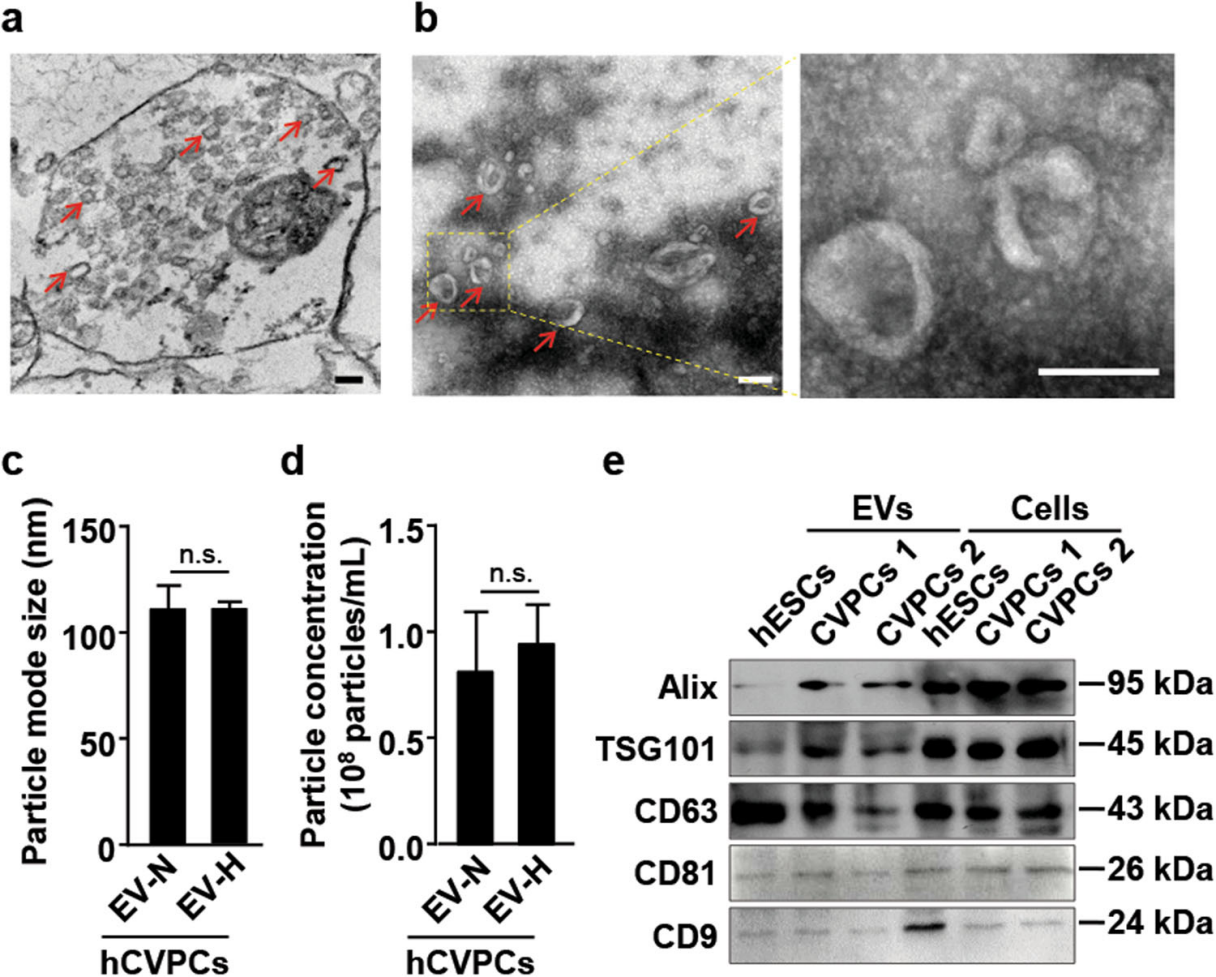
Identification of extracellular vesicles (EVs) secreted by hESC-CVPCs. **a** Transmission electron microscope revealed that hESC-CVPCs contain multivesicular bodies (MVBs). **b** Extracellular vesicles isolated from hESC-CVPC-conditioned medium showed typical cup-shaped shapes at diameter ~30–150 nm. **c**, **d** The nanoparticle tracking analysis of the mode size **c** and the particle concentrations for the EVs secreted from hCVPCs with (EV-H) and without hypoxia treatment (EV-N) in the culture supernatant of hCVPCs (**d**). *n* = 5. **e** Western blot analysis of the EV markers, Alix, TSG101,CD63, CD9, and CD81. Scale bar, 100 nm.

SSEA1^+^-hCVPCs were generated from hESC line H9 (WiCell) as previously reported^21,25,26,45^. The generated cells expressed SSEA1, a surface marker of hCVPCs^57,58^, in 96.8–97.8% purity analyzed by flow cytometry (Supplementary Fig. S3a) and displayed early CVPC markers MESP1, ISL1, MEF2C, GATA4, and NKX 2-5 detected by immunostaining (Supplementary Fig. S3b). Transmission electron micrographs of hCVPCs demonstrated the presence of EV-like vesicles within multivesicular bodies (MVBs) in the cytoplasmic area (Fig. 1a). The secreted EVs were isolated from hCVPCs and showed a double-membrane-bound, cup-shaped typical shape (Fig. 1b). Nanoparticle tracking analysis (NTA) confirmed the mode size of secreted EVs from hCVPCs was around 118 nm in the EV-N and 110 nm in the EV-H (Fig. 1c), with the particle concentrations around 0.82 × 10^8^/mL in the original hCVPC supernatant and 0.95 × 10^8^/mL in the hypoxia-treated hCVPC supernatant (Fig. 1d). The Western blot analysis further confirmed the EV marker proteins ALG-2 interacting protein X (ALIX), tumor susceptibility gene 101 protein (TSG101), CD63, CD9, and CD81 in the hESCs, hCVPCs and the EV preparations derived from these cells (Fig. 1e). These data demonstrate the successful isolation of hCVPC-secreted EVs.

### Intramyocardial delivery of hCVPC-EVs improves post-MI cardiac function and reduces scar size

**Fig. 2:**
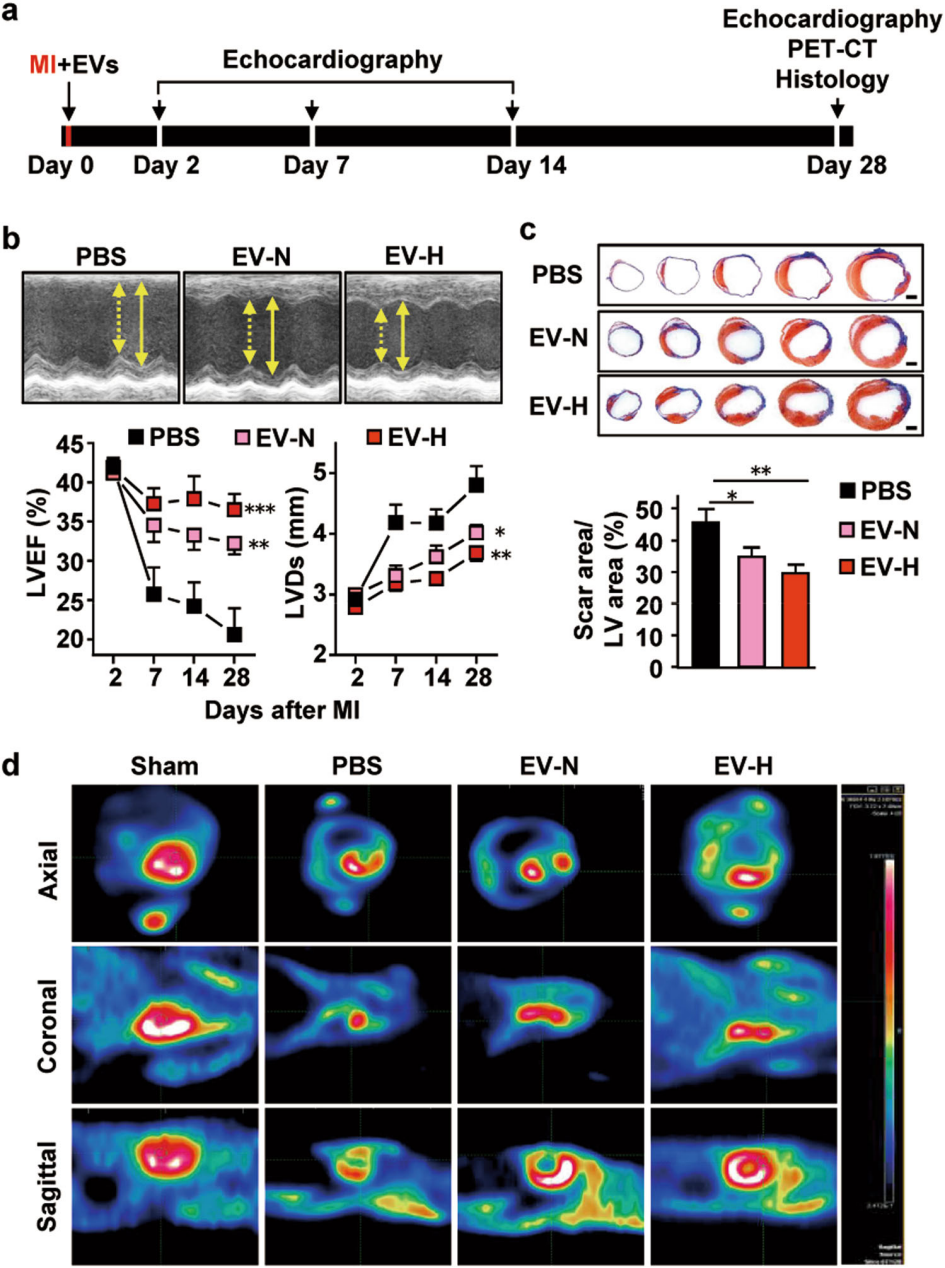
Cardioprotective effects of hCVPC-EVs delivered to acutely infarcted murine hearts by permanent ligation of the LAD coronary artery. **a** Schematic of treatment and analysis using hCVPC-extracellular vesicles. **b** Echocardiographic analysis of LVEF and LVDs. *n* = 8 (PBS), 8 (EV-N), and 11 (EV-H). **c** Representative cross-sectional images and quantitative data of hearts stained with Masson’s trichrome at day 28 post MI. *n* = 6–8. Scale bars, 1 mm. **d** In vivo PET/CT images at day 28 post MI. *p < 0.05, **p < 0.01, ***p < 0.001.

To assess the therapeutic efficacy of hCVPC-EVs when delivered at the acute phase of the murine MI model, the EVs secreted from hCVPCs under normoxic (EV-N) and hypoxic (EV-H) culture conditions were intramyocardially injected in mice with AMI. The functional outcome was assessed at 2, 7, 14, and 28 days post MI as indicated in Fig. 2a. The establishment of MI model by permanent ligation of the LAD coronary artery was confirmed by echocardiography analysis (Fig. 2b). Changes in LVEF and LVDs (Fig. 2b) were similar among the vehicle PBS control, EV-N and EV-H groups at day 2 post MI, indicating a comparable infarcted area among the groups when the MI model was undertaken. However, MI-worsened LVEF and LVDs in the PBS control group were significantly improved in the EV-N and EV-H groups during the observation time up to 28 days post MI, with the EV-H showed a better recovery tendency (Fig. 2b). Consistently, Masson’s trichrome-staining analysis showed that the scar area/LV area was significantly smaller in the EV-N and EV-H groups than that in the PBS group at day 28 post MI (Fig. 2c). Micro-PET analysis further showed the uniform uptake of fludeoxyglucose (FDG), demonstrating normal myocardial viability^59^ in the heart of Sham mouse, a smaller FDG-uptake area in the MI heart, and partially restored areas in the EV-N and EV-H groups at day 28 post MI (Fig. 2d). Taken together, these results demonstrate that the hCVPC-secreted EVs have cardioprotective effects in augmenting cardiac function and limiting fibrosis formation. Moreover, the EVs secreted from hCVPCs under the hypoxia-culture condition appear to have a better benefit in improving cardiac function of the infarcted hearts.

### hCVPC-EVs improve cardiomyocyte survival in vivo and in vitro

**Fig. 3:**
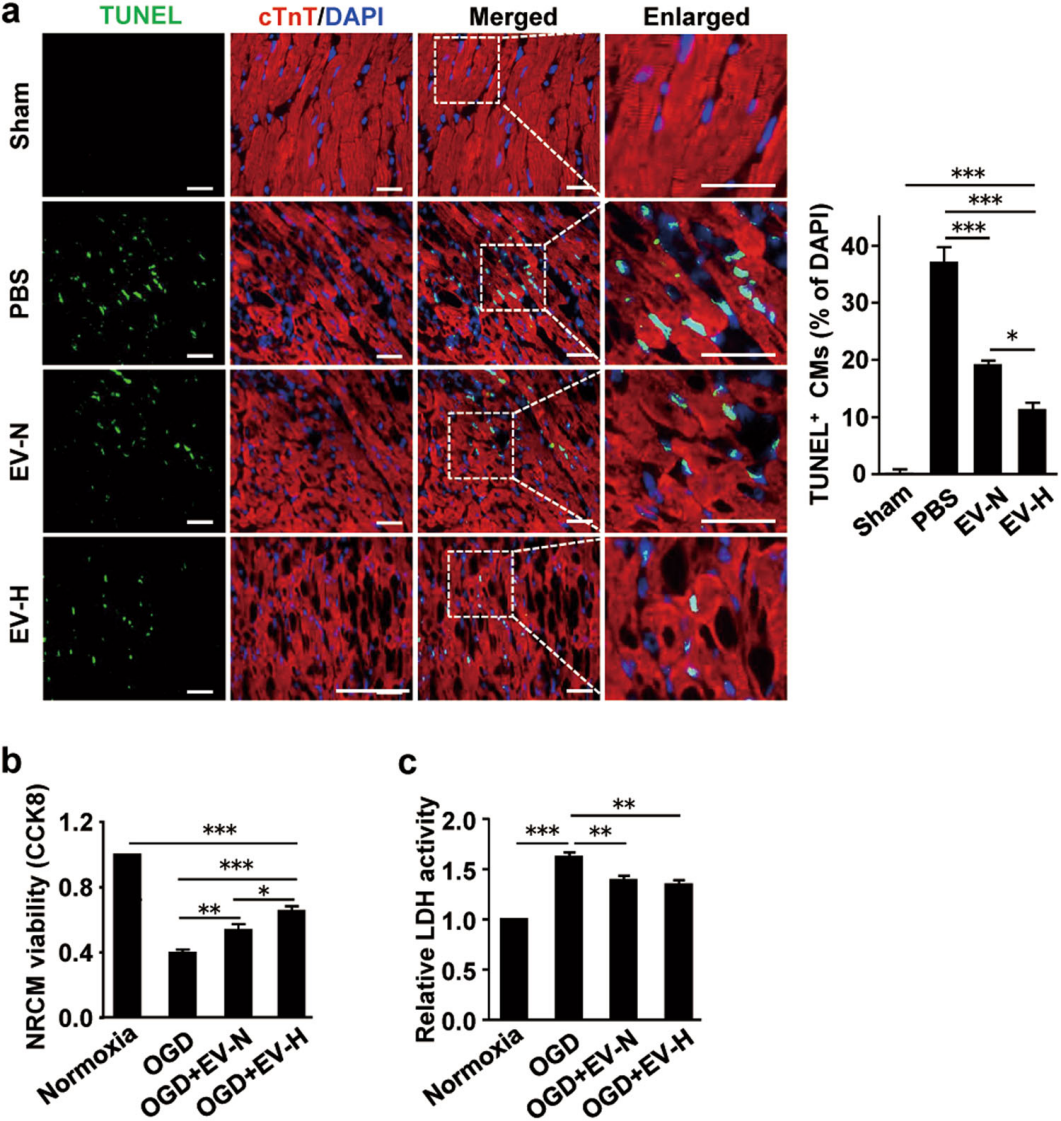
hCVPC-extracellular vesicles reduce cell death of cardiomyocyte. **a** Representative and quantification of IHC staining for TUNEL^+^ cells in the border zone of infarcted hearts at day 3 post MI and sham hearts. *n* = 12 slices from 4 hearts each group. Scale bar, 100 μm. **b** Cell viability of NRCMs under OGD injury treated with the EV-N and the EV-H, *n* = 6. Scale bar, 100 μm. **c** The activity of LDH in the culture medium of NRCMs subjected to a 6-h of OGD injury with and without the treatment of 1 μg EV-N or EV-H. *n* = 5. *p < 0.05, **p < 0.01, ***p < 0.001. cTnT cardiac troponin T, TUNEL terminal deoxynucleotidyl transferase dUTP nick end labeling, OGD oxygen glucose deprivation.

To determine whether the inhibition of cardiomyocyte death is one of mechanisms underlying the cardioprotective effects of hCVPC-EVs, we examined the number of TUNEL^+^-cardiomyocytes in the infarcted hearts. The TUNEL^+^ cardiomyocytes in the border zone of day 3 post MI hearts were significant reduced in the EV-N and EV-H groups and, notably, the number of TUNEL^+^ cardiomyocytes in the EV-H group were lesser than that in the EV-N (Fig. 3a), indicating that the EVs secreted from hypoxia-conditioned hCVPCs have a better effect on the inhibiting of cardiomyocyte death. These findings suggest that hCVPC-EVs delivery can protect cardiomyocytes from MI-induced cell death, and this effect can be enhanced by hypoxia-conditioning of hCVPCs.

To determine the direct effect of hCVPC-EVs on the cardiomyocytes, we examined the role of hCVPC-secreted EVs in the NRCMs with the OGD injury. Consistent with the in vivo data, the OGD treatment significantly reduced the cell viability, while the reduction was significantly attenuated in the EV-N- and the EV-H-treated cells, and the latter showed a better protective effect than that in the EV-N (Fig. 3b). In addition, the OGD-enhanced LDH activity in the culture medium of NRCMs was significantly reduced in the EV-N and EV-H groups (Fig. 3c). These results suggest that both EV-N and EV-H can improve the survival of cardiomyocytes during in vivo MI and in vitro OGD injury.

### hCVPC-EVs promote angiogenesis in vivo and in vitro

**Fig. 4:**
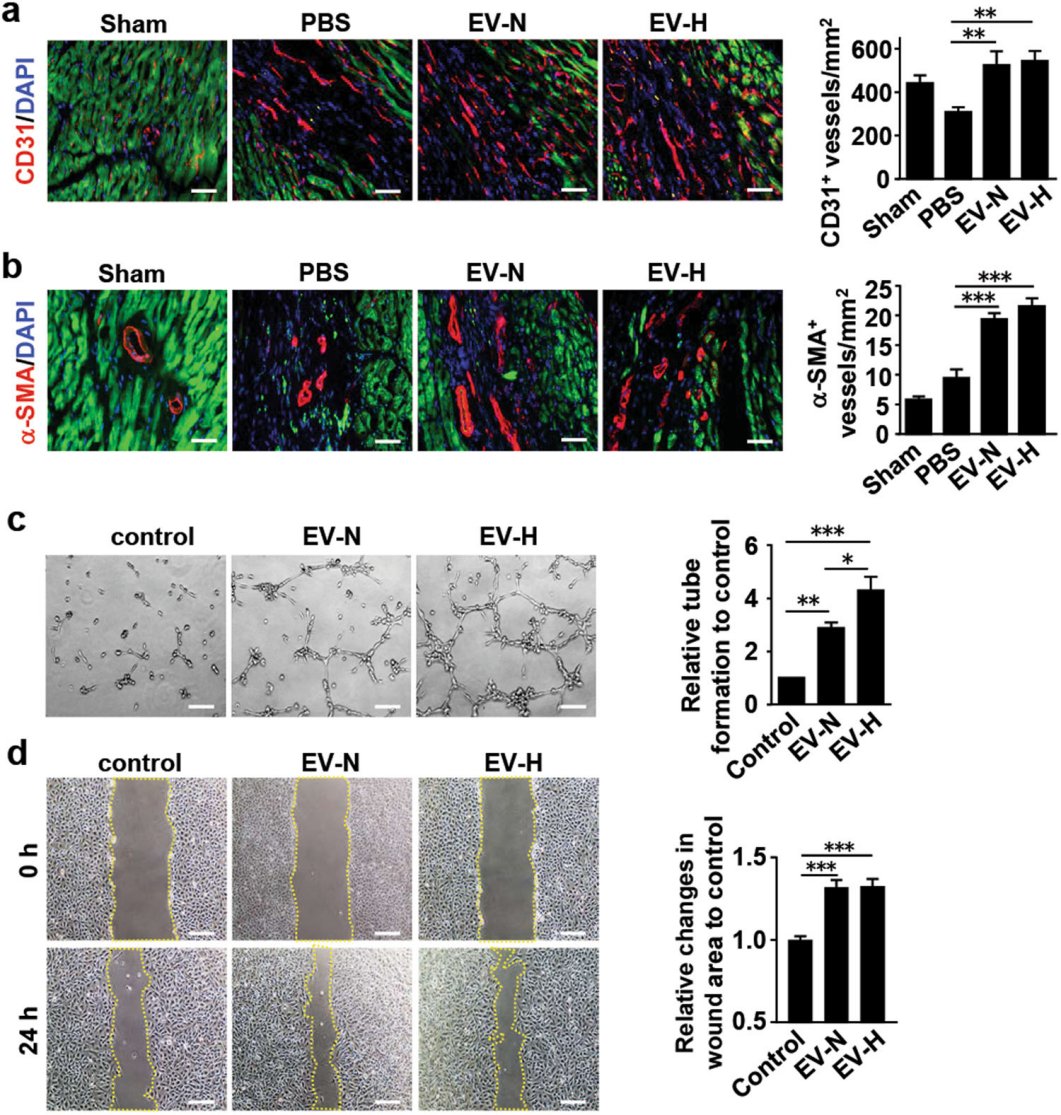
Effects of hCVPC-EVs on HUVECs migration, tube formation and NRCMs protection. **a**, **b** Representative and quantification of immunohistochemical (IHC) staining for CD31^+^ endothelial cells (**a**) and α-SMA^+^ blood vessels (**b**) in the border zone of infarcted hearts at day 28 post MI. *n* = 12 slices from 4 hearts each group. **c** Tube formation of HUVECs treated with EV-N and EV-H (*n* = 6). **d** Migration of HUVECs treated with the EV-N and the EV-H after inhibition of proliferation with 10 μg /mL mitomycin for 2 h (*n* = 5). Scale bar, 100 μm. *p < 0.05, **p < 0.01, ***p < 0.001.

Angiogenesis can also contribute to the reduction of cell death and fibrosis size in the infarcted hearts. We therefore examined the blood vessel density in the infarcted hearts. Immunohistological chemistry analysis showed that the number of CD31^+^ vessels (Fig. 4a) and α-SMA^+^ vessels (Fig. 4b) at the border zone of day 28 post MI hearts was significantly increased in the EV-N and EV-H groups compared with these in the PBS control ones. The effect of hCVPC-EVs on endothelial cells was further examined by tube-formation assay using HUVECs. The tube length of HUVECs treated with hCVPC-EV-N or hCVPC-EV-H for 8 h was significantly increased compared with that in the PBS control group, with a better effect in the hCVPC-EV-H than that in the hCVPC-EV-N (Fig. 4c). The HUVEC migration after the inhibition of proliferation by 10 μg/mL mitomycin was promoted in the hCVPC-EV-treated groups (Fig. 4d). These data demonstrate that the beneficial effect of hCVPC-EVs in endothelial cells may contribute to the promoted angiogenesis seen in the hCVPC-EV-treated infarcted hearts.

### Identification of RNAs in the hCVPC-EVs

**Fig. 5:**
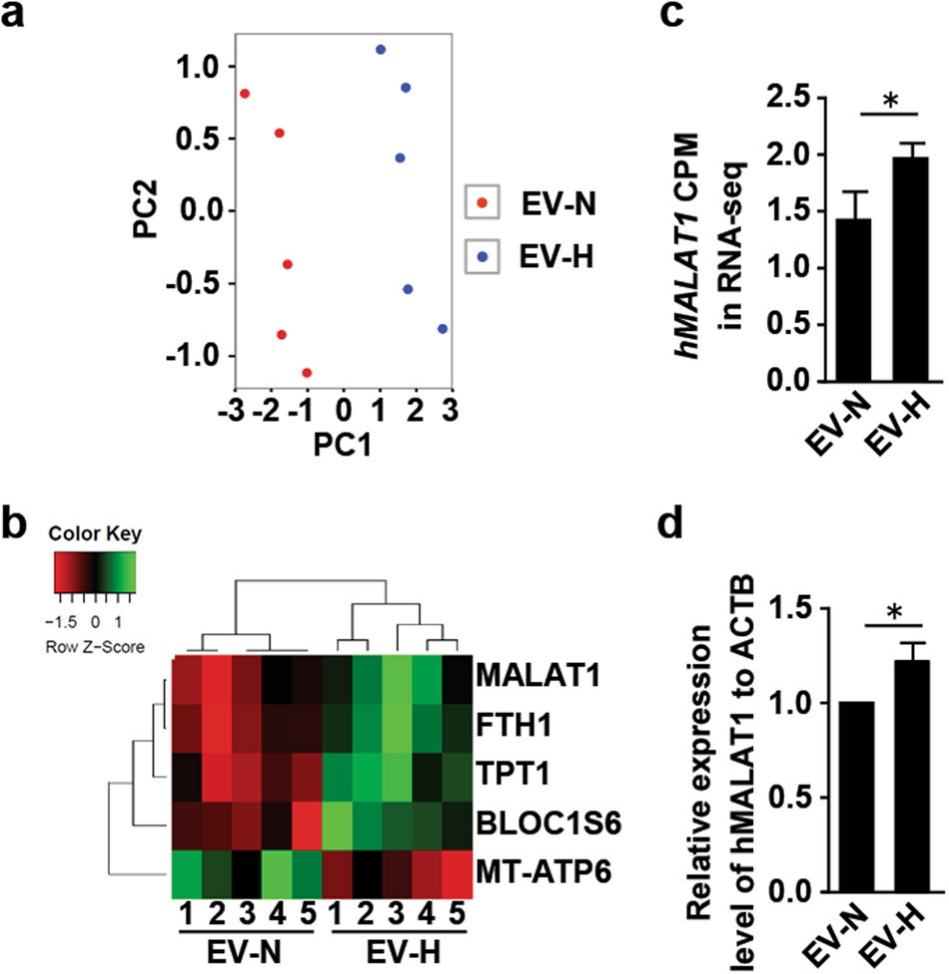
The analysis of total RNAs in hCVPC-EVs. **a**, **b** EV-N and EV-H samples were shown to segregate in PCA (**a**), and based significantly on the expression of five genes shown by hierarchical clustering (**b**). **c** The CPM values of the sequencing results showed that MALAT1 abundance in EV-H was significantly higher than that in EV-N. **d** MALAT1 expression was verified by RT-qPCR. *n* = 5. *p < 0.05. CPM counts per million, PCA principle component analysis.

To understand the mechanism of the cardioprotective effect of hCVPCs-Evs, we performed RNA-seq analysis on the EVs isolated from hCVPCs with or without hypoxia-conditioning and then aligned them to a database for RNA annotations (Fig. 5a). Some mRNAs with known functions were detected in both EV-N and EV-H groups, such as insulin like growth factor 2 (IGF2) and superoxide dismutase 2 (Sod2), which were reported to improve cardiomyocyte survival^47,60^. Similarly, ferritin heavy chain 1 (FTH1) and endothelial PAS domain protein 1 (EPAS1) mRNA detected in the EV-N and EV-H have also been shown to promote angiogenesis^61–63^. The differential analysis using corrected counts per million (CPM) identified five genes with significant changes between the EV-N and the EV-H groups that were segregated by hierarchical clustering (Fig. 5b). Among them, four mRNAs, FTH1, TPT1, BLOC1S6, MT-ATP6, and one lncRNA MALAT1 were differentially expressed between the two groups (Fig. 5b). The CPM values of the sequencing results showed that MALAT1 abundance in the EV-H was significantly higher than that in the EV-N (Fig. 5c). The differential expression for MALAT1 was further confirmed by RT-qPCR analysis (Fig. 5d). These data suggest that the MALAT1 seems exist in the hCVPC-EVs and might be involved in the beneficial effects of the hCVPC-EVs in the cardioprotection.

### LncRNA MALAT1 in the hCVPC-EVs contributes to cardiomyocyte protection and tube formation

**Fig. 6:**
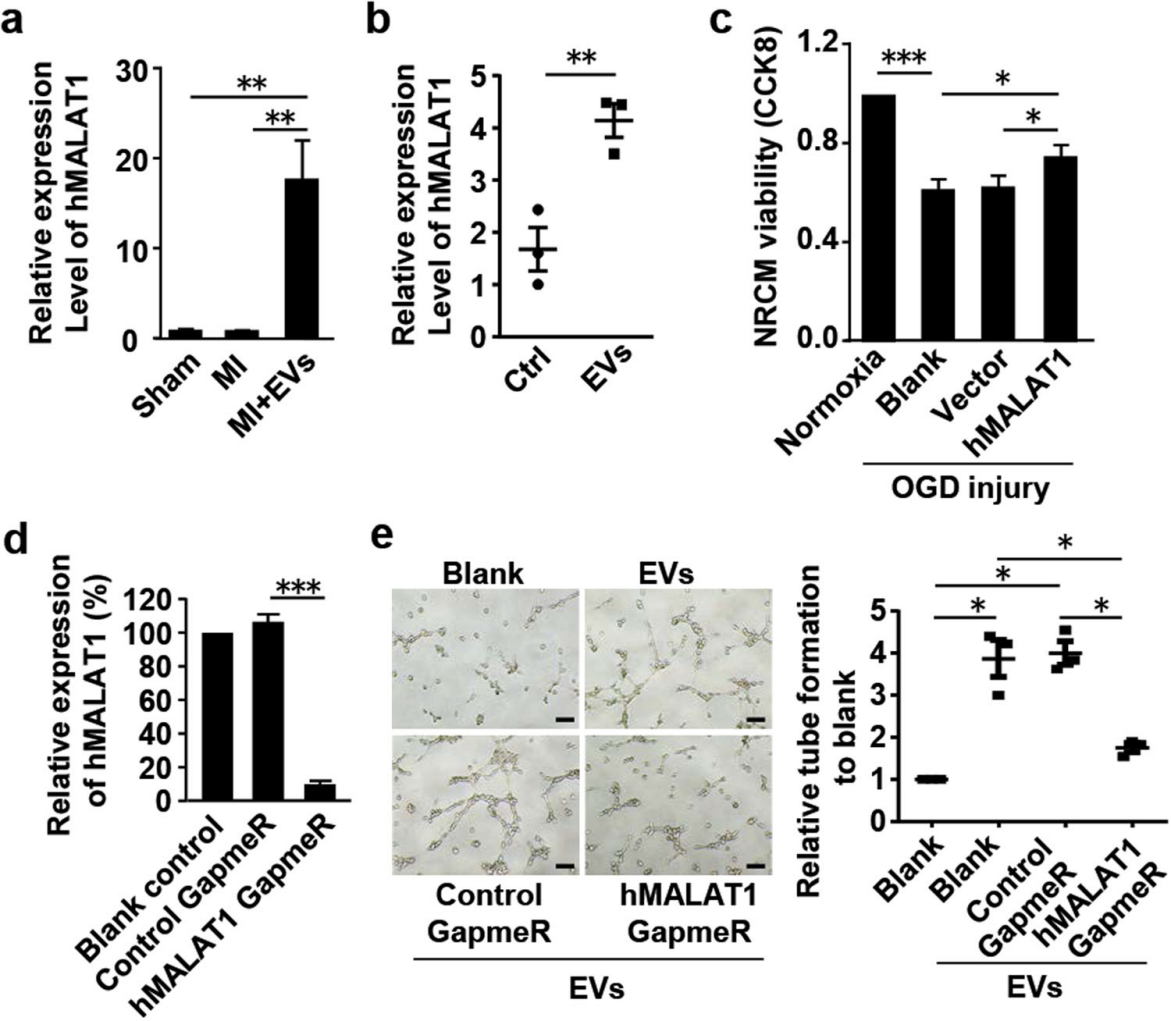
MALAT1 promotes HUVEC tube formation and cardiomyocyte survival. **a** The abundance of exogenous MALAT1 was significantly increased in cardiac tissue of the MI + EV group (*n* = 7) compared with the sham group (*n* = 4) and the MI group (*n* = 5). **b** The MALAT1 abundance was significantly upregulated in the NRCMs treated with hCVPC-EVs. *n* = 3. **c** Cell viability in the MALAT1-overexpressing NRCMs with and without OGD injury. *n* = 9. **d** MALAT1 was knocked down in the HUVECs with the MALAT1 GapmeR. *n* = 5. **e** MALAT1 GapmeR inhibited hCVPC-EV-promoted HUVECs tube formation *n* = 3 each. Scale bar, 100 μm. *p < 0.05, **p < 0.01, ***p < 0.001.

To determine whether MALAT1 contributes to cardiomyocyte survival and tube formation of endothelial cells, we performed experiments with knockdown and overexpression of hMALAT1. The hMALAT1 abundance was significantly upregulated in the MI myocardium injected with the hCVPC-EVs compared with these in the Sham and the MI groups (Fig. 6a). Similarly, the hMALAT1 abundance was enhanced in the NRCMs (Fig. 6b) and the AMCMs (Supplementary Fig. S4) treated with the hCVPC-EVs. Overexpression of hMALAT1 was confirmed to improve the viability of NRCMs during OGD injury (Fig. 6c). In addition, the hMALAT1 was knocked down by ~90% with MALAT1 GapmeR in the recipient HUVECs (Fig. 6d). The hCVPC-EV-promoted tube formation of HUVECs was significantly inhibited by the knockdown of hMALAT1 with hMALAT1 GapmeR (Fig. 6e).

### MALAT1 exerts cardioprotective effects by targeting miR-497

**Fig. 7:**
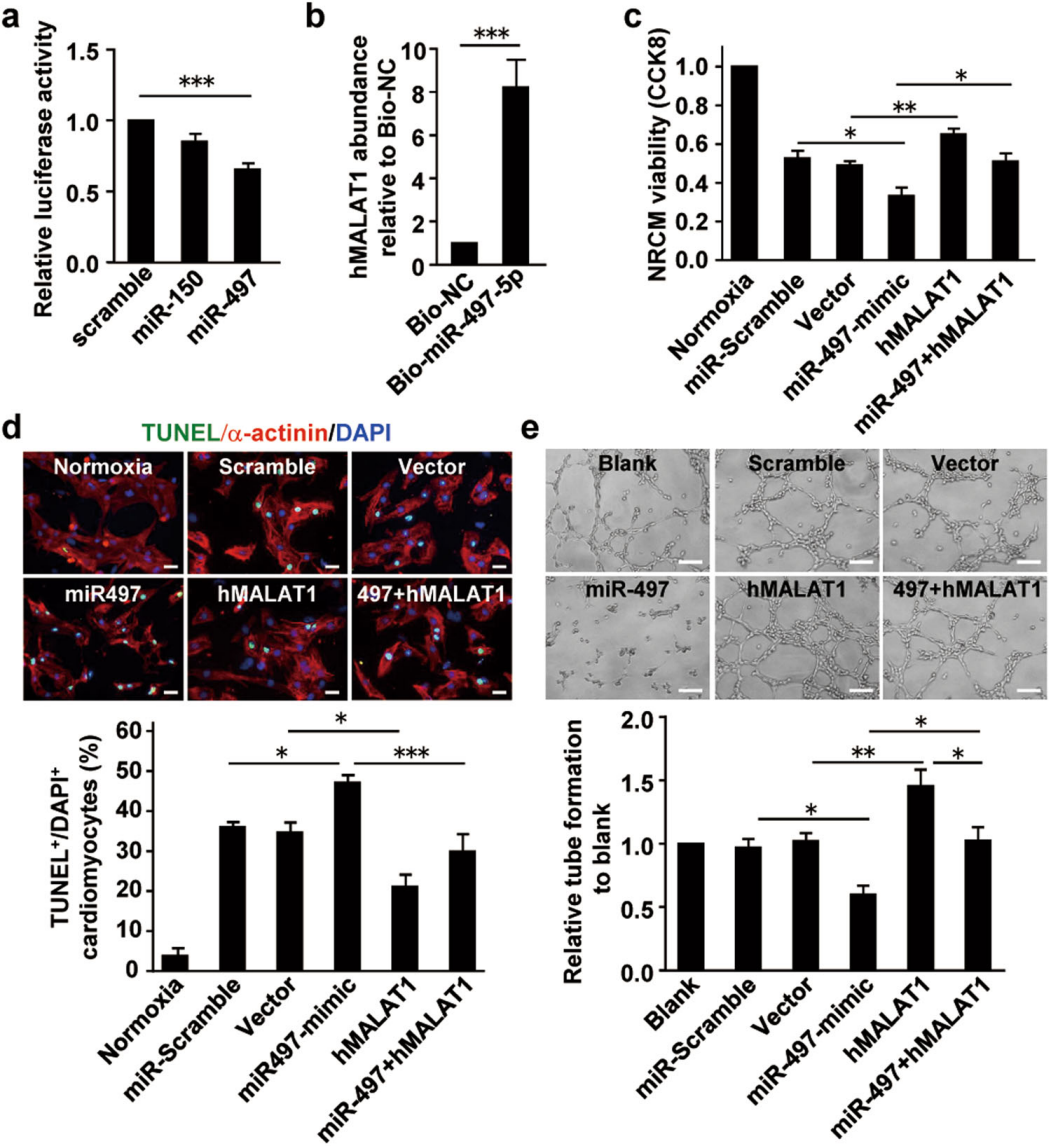
MALAT1 cancels the pro-cardiomyocyte-death effect and suppress-angiogenesis effect of miR497. **a** Luciferase activity of psiCHECK2-MALAT1 upon transfection of indicated miRNA mimics in 293T cells. **b** The RNA pull down assay in the HUVECs transfected with biotin-labeled miR-497-5p (Bio-miR-497-5p). The MALAT1 combined with Bio-miR-497-5p was pulled down with Pierce^™^ Streptavidin Magnetic Beads. **c** The cell viability in the NRCMs under OGD injury after transfection of pcDNA3.1-MALAT1 and/or miR-497. **d** Representative and quantification of IHC staining for TUNEL^+^ NRCMs after OGD injury. **e** HUVEC tube formation after the transfection of pcDNA3.1-MALAT1 and/or miR-497. TUNEL terminal deoxynucleotidyl transferase dUTP nick end labeling, OGD oxygen glucose deprivation. Scale bar, 100 μm. *n* = 5 each for (**a**–**e**). *p < 0.05, **p < 0.01, ***p < 0.001.

Next, we investigated the potential molecular target of MALAT1 in the cardioprotection. The potential binding of MALAT1 to miR-497 was predicted by using miRcode Website (mircode.org) and DianaTools Website (http://carolina.imis.athena-innovation.gr/diana_tools/web/index.php?r=lncbasev2%2Findex-predicted). The analysis showed that there were six predicted binding sites of MALAT1 with miR-497 (Supplementary Fig. S5). The expression of miR-497 was detected in the mouse ventricles of Sham, MI + PBS, and MI + hCVPC-EV groups (Supplementary Fig. S6a) as well as in the NRCMs (Supplementary Fig. S6b). To confirm it, we constructed luciferase vectors carrying the hMALAT1 cDNA full length sequence to examine the potential direct bindings between the MALAT1 and miR-497, and miR-150 was used as a negative control. As shown in Fig. 7a, the luciferase activity was significantly decreased in the miR-497- but not the miR-150-mimic transfected cells compared with that of the control-mimic transfected cells. The RNA pull down assay further confirmed that the hMALAT1 was enriched about the 8.2-fold in the Bio-miR-497-5p group (biotin-labeled miR-497-5p) compared with the control group (Bio-NC, biotin labeled scramble RNA) in the HUVEC, indicating that MALAT1 could bind with miR-497-5p (Fig. 7b). Next, we examined the function of MALAT1 and miR-497 on cardiomyocytes and endothelial cells. In NRCMs, OGD injury-induced reduction of cardiomyocyte viability (Fig. 7c) and increased TUNEL^+^ cardiomyocytes (Fig. 7d) were aggravated by miR-497-mimic and the protective effects of MALAT1 were also reversed by miR-497-mimic. Similarly, the tube formation of HUVECs was impaired by miR-497-mimic and MALAT1 promoted tube formation was also abrogated by miR-497-mimic (Fig. 7e). These results indicate that MALAT1 from hCVPC-EVs may exert its cardioprotective effects by targeting miR-497.

## Discussion

**Fig. 8:**
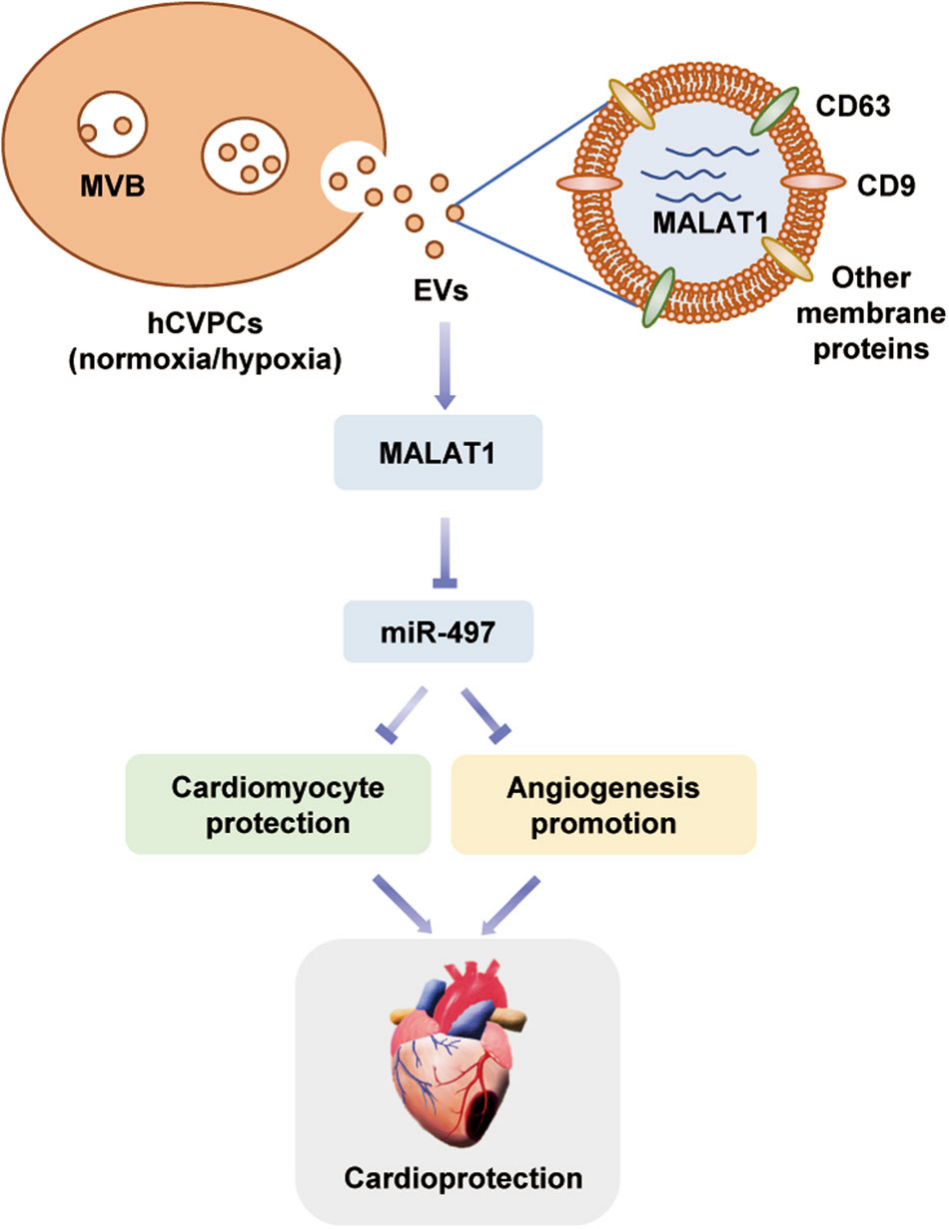
Schematic representation of the therapeutic effect of hCVPC-secreted extracellular vesicles in the infarcted hearts. Delivery of hCVPC-EVs during early phase of MI has many beneficial effects of promoting cardiac function, reducing fibrosis, improving cardiomyocyte survival, and promoting angiogenesis. These beneficial effects are enhanced by using the extracellular vesicles secreted from hypoxic hCVPCs. Moreover, the hCVPC-EVs contain lncRNA MALAT1, which appear to contribute to the inhibition of cardiomyocyte death and promoting of angiogenesis via targeting miR-497, and the MALAT1 abundance in the hCVPC-EVs can be up-regulated by hypoxia-conditioning of hCVPCs.

In this study, we report that the hESC-CVPC-secreted EVs protect acutely infarcted hearts from progressive worsening of cardiac function and reduce scar formation when delivered at the early phase of MI. Our results point to the protective effects being associated with the improvement in the existing cardiomyocyte survival and vascularization. Further analysis shows that the cardioprotective effects of hCVPC-EVs appear to be partially mediated by the hCVPC-EV-contained lncRNA MALAT1 via targeting miR-497 and the abundance of MALAT1 in the hCVPC-EVs is upregulated in hypoxia-treated hCVPCs (Fig. 8). These findings reveal the previously unrecognized role of the hCVPC-secreted EVs in cardiac protection when given during the acute phase of MI and provide new insights into the RNA content of hCVPC-EVs and how a lncRNA mediator exerts cardioprotective effects.

EVs secreted from stem cells and hPSC-derived cardiac lineage cells hold tremendous promise for cardioprotection, while the secreted EVs are not equal among cells^9,30,40,64,65^. Here, we demonstrate for the first time that the hCVPC-EV delivery administrated at the acute phase of MI improves cardiac function and reduces scar tissue, which recapitulates the beneficial effects of their parent cells in the treatment of AMI in the murine^25^ and nonhuman primate model^25,26^. These findings, together with the observations of the cardioprotective effects from various stem cells and their secreted EVs when delivery at the acute phase of MI^39,40,65–68^ or during early reperfusion^69^, indicate the acute phase of MI is a critical window for promoting infarct healing.

Our findings are consistent with the observation of the improvement of cardiac function when the delivery of EVs either secreted from SSEA1^+^-hESC- or hiPSC-derived CVPCs (hCVPC-EVs) into the murine hearts after 2–3 weeks of post-MI chronic HF^32,33^. Moreover, the hCVPC-EVs recapitulate the beneficial effects of their parent cells in the treatment of either AMI^25,26^ and chronic post-infarct HF murine model, suggesting that the EVs are active elements of secretome of hPSC-derived cardiac lineage cells, playing major roles in the salvage of infarcted myocardium. Similar observations are obtained from the EVs secreted by hPSC-derived cardiomyocytes^41,65,68,70^. Considering the allogeneic EVs do not induce significant immune responses of the parent cells after repeated dosing^30^, EVs secreted by stem cells and hPSC-derived cardiovascular cells might be used as therapeutic agents, and these vesicles need to be thoroughly dissected to explore the potential use of a cell-derived but cell-free therapy for cardioprotection.

Another finding here is that the EVs from hypoxic hCVPCs have better benefits in the improvement of cardiomyocyte survival and the promotion of vascularization than these in the normoxic ones. Similar results showing the enhanced cardioprotective effects from the EVs secreted by hypoxic c-kit^+^ cells^69^ and MSCs^13^ were observed in a rat model of I/R and a AMI model of nonhuman primates. These observations suggest that the secretome of implanted stem/progenitor cells would alter in the postinfarct tissue and a certain degree of hypoxia-preconditioning of the cells before the implantation would be a way to enhance the therapeutic effect of both cells and secreted vesicles.

It has been shown that the EVs communicate with the target cells directly and transfer the RNAs and proteins representing their parental cells^37,51,71^. Cumulated evidence has shown that the miRNAs in the stem cell-secreted EVs contribute critically to the vesicle-mediated benefits for cardioprotection^40,69,72^. The hCVPC-EVs have been shown containing the specific miRNA signature associated with the tissue-repair pathways^33^, while little is known whether lncRNAs in the EVs from hPSC-derived cardiac lineage cells. Here, we identified the existence of lncRNA MALAT1 in the hCVPC-EVs. This is supported by the following observations: (i) the MALAT1 is detected in the RNAseq analysis from the EVs secreted by hCVPC-EV-N and its abundance is higher in the EVs secreted from hypoxic treated hCVPCs than in the hCVPC-EV-N (Fig. 5a–c); (ii) these results are confirmed by RT-qPCR analysis (Fig. 5d); (iii) the high abundance of exogenous hMALAT1 is detected in the cardiac tissue of the infarcted hearts injected with hCVPC-EVs compared with that in the Sham group and the MI group (Fig. 6a); and (iv) the hMALAT1 abundance is significantly upregulated in the cultured NRCMs (Fig. 6b) and the AMCMs (Supplementary Fig. S4) treated with the hCVPC-EVs. Supportively, the lncRNAs are detected in the EVs secreted from hESC/iPSC-derived cardiomyocytes^41^.

We further confirmed that the MALAT1 protects the cardiomyocytes from OGD injury and promotes tube formation of endothelial cells. These effects may partially contribute to the cardioprotection of hCVPC-EVs as EVs contain many kinds of nucleic acids and proteins. Consistently, MALAT1 has been detected in the EVs from human umbilical cord MSCs and shown to prevent aging-induced cardiac dysfunction^44^. It also contributes the vascular growth in mouse retinal angiogenesis and hindlimb ischemia model^43^. Other kinds of cardioprotective components need to be investigated in further research.

Mechanistically, we identified that MALAT1 exerts its beneficial effects through targeting miR-497. This is supported by the following observations: (i) the luciferase activity assay suggests that miR-497 might be a target of MALAT1 (Fig. 7a); (ii) the miR-497-mimic aggravates the cardiomyocyte injury and canceled the protective effect of MALAT1 in the cardiomyocytes (Fig. 7c, d); (iii) MALAT1-promoted the tube formation of endothelial cells is abrogated by miR-497 (Fig. 7e); and (iv) the RNA pull down assay showed that MALAT1 has an interaction with miR-497 in the HUVECs (Fig. 7b). These data reveal a new target of MALAT1 in the cardioprotection. The deteriorative effects of miR-497 in cardiomyocytes is consistent with the observation showing that the inhibition of miRNA-497 ameliorates anoxia/reoxygenation-induced cardiomyocyte injury by suppressing cell apoptosis and enhancing autophagy^73^. Although our results identify the binding of MALAT1 to miR-497, it is unclear how many MALAT lncRNA need to be delivered to infarcted hearts to sufficiently bind and inhibit miR497. Taken together, the MALAT1 in hCVPC-EVs appears to be one of the mechanisms partially contribute to the cardioprotective effect of hCVPC-EVs via promoting angiogenesis and inhibiting cardiomyocyte death by targeting miR-497. The effects of MALAT1 and miR-497 in the infarcted hearts need to be identified and the mechanisms of hCVPC-EVs in cardioprotection are required to be thoroughly elucidated.

*Limitations*: The cardiomyocytes we used for the in vitro injury model are NRCMs (Fig. 7), which do not always represent the response in the adult cardiomyocytes. The cardioprotective effects of MALAT1 and the underlying mechanisms need to be verified in the infarcted hearts.

In conclusion, we have shown that the delivery of hCVPC-EVs during early phase of AMI can promote recovery of cardiac function and reduce fibrosis formation. These beneficial effects are enhanced by using the EVs secreted from hypoxia-treated hCVPCs and are associated with the improvement of existing cardiomyocyte survival at the acute phase of infarcted hearts and the promotion of angiogenesis. Moreover, the hCVPC-EVs contain lncRNA MALAT1, which seems partially contribute to the inhibition of cardiomyocyte death and promotion of angiogenesis via targeting miR-497. In addition, the MALAT1 abundance in the hCVPC-EVs can be upregulated by hypoxia-conditioning of hCVPCs. These findings provide new insights into the cardioprotective mechanisms of hCVPCs and the cell-secreted EVs, and suggest that hCVPC-EVs might be used as a tool to understand the mechanism of infarct healing and promote healing of infarcted hearts.

## Supplementary information

CDDIS-19-3299RR Supplementary Figure Legends

Supplementary Figure S1

Supplementary Figure S2

Supplementary Figure S3

Supplementary Figure S4

Supplementary Figure S5

Supplementary Figure S6
