# Supplementary material for: Extracellular vesicles from human embryonic stem cell-derived cardiovascular progenitor cells promote cardiac infarct healing through reducing cardiomyocyte death and promoting angiogenesis
Source: Cell Death Dis. 2020 May 11;11(5):354. doi: 10.1038/s41419-020-2508-y (PMC7214429; doi:10.1038/s41419-020-2508-y)
Supplement: Supplementary file 1 — CDDIS-19-3299RR Supplementary Figure Legends [file 41419_2020_2508_MOESM1_ESM.docx]

**Supplementary Figure Legends**

**Supplementary Fig. S1 Relationship between the protein concentration and the number of particles secreted from the hCVPCs with (EV-H) and without hypoxia treatment (EV-N) based on the data from NTA and Pierce BCA Protein Assay.** n.s., no significant statistical difference. n = 5 each.

**Supplementary Fig. S2 The map of psiCHECK2-MALAT1 plasmid.** The hMALAT1 cDNA full length (8779 bp) was cloned into the psiCHECK2 (Promega, Madison, WI) from the pcDNA3.1(-)-MALAT1 plasmid (Shanghai Integrated Biotech Solutions Co., Ltd) between the XhoI and NotI restriction endonuclease sites.

**Supplementary Fig. S3 Characterization of human embryonic stem cells (hESCs)-derived cardiovascular progenitor cells (CVPCs). a** Differentiated hESC-CVPCs that expressed SSEA1 were evaluated via flow cytometry. n = 5; **b** Differentiated hESC-CVPCs were immunostained for expression of the CVPC markers MESP1 (mesoderm posterior BHLH transcription factor 1), ISL1 (ISL LIM homeobox 1), MEF2C (myocyte enhancer factor 2C), GATA4 (GATA binding protein 4), and NKX2-5 (NK2 homeobox 5). Similar observations were obtained from more than 5 independent experiments. Bar = 50 μm.

**Supplementary Fig. S4 Q-PCR analysis of the hMALAT1 level with and without hCVPC-EV treatment in the adult mouse cardiomyocytes (AMCMs).** The high abundance of exogenous hMALAT1 was detected in the AMCMs treated with hCVPC-EVs (1 μg/mL) for 24 hours but not in the PBS control group. n = 5 each. **p < 0.01.

**Supplementary Fig. S5 The binding site prediction of MALAT1 with miR-497 by DianaTools Website.** Six binding sites of MALAT1 with miR-497 were predicted (<http://carolina.imis.athena-innovation.gr/diana_tools/web/index.php>).

**Supplementary Fig. S6 Q-PCR analysis of the miR-497 level in the mouse myocardium and the NRCMs.** The expression of miR-497 was detected in the mouse ventricles of Sham, MI, and hCVPC-EV groups (**a**) and in the NRCMs (**b**). The experimental number is indicated on the figures. n.s., no significant statistical difference.
