# Supplementary figures and images for: Extracellular vesicles from human embryonic stem cell-derived cardiovascular progenitor cells promote cardiac infarct healing through reducing cardiomyocyte death and promoting angiogenesis
Source: Cell Death Dis. 2020 May 11;11(5):354. doi: 10.1038/s41419-020-2508-y (PMC7214429; doi:10.1038/s41419-020-2508-y)

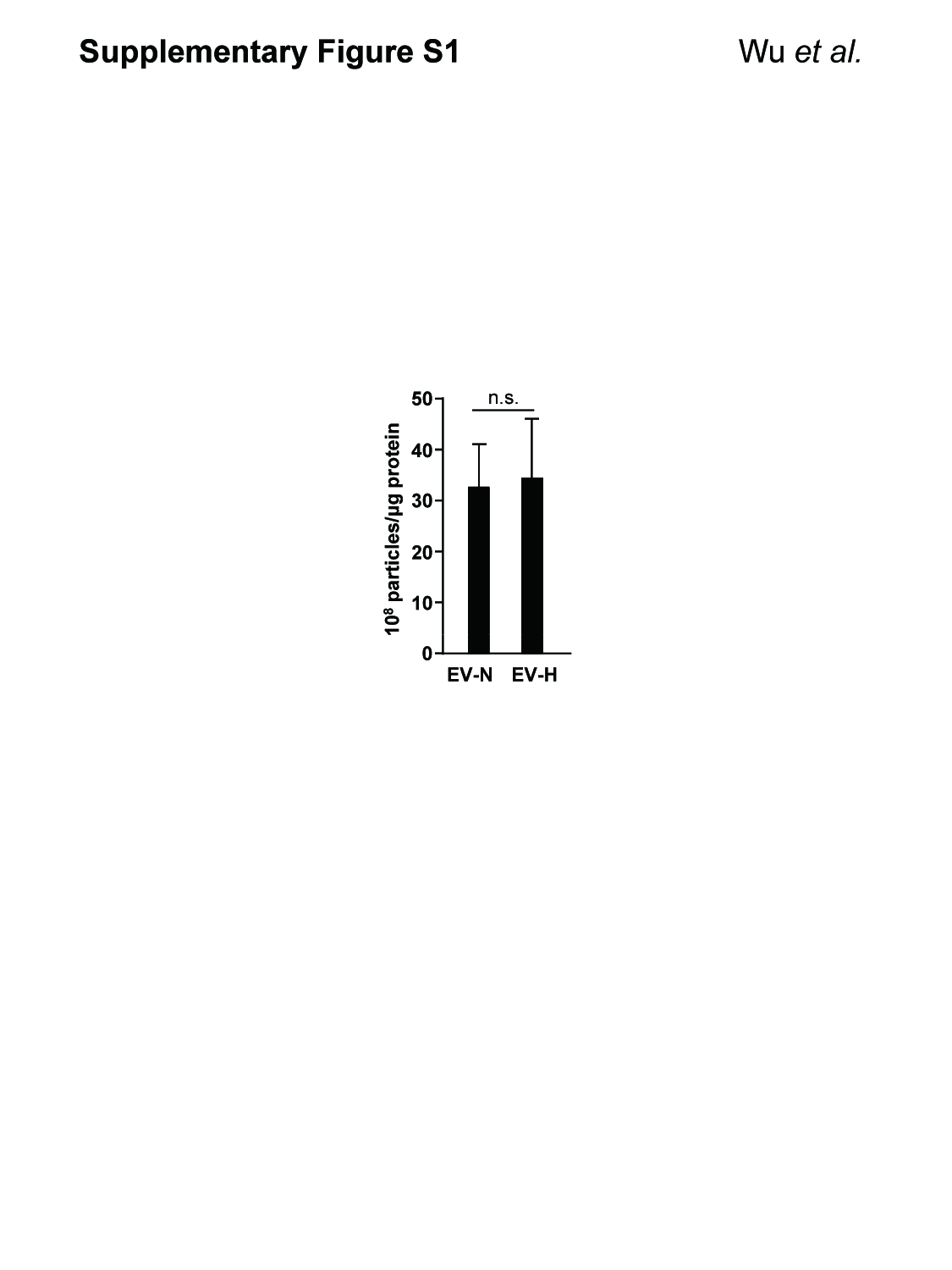

Supplement: Supplementary file 2 — Supplementary Figure S1 [file 41419_2020_2508_MOESM2_ESM.tif]

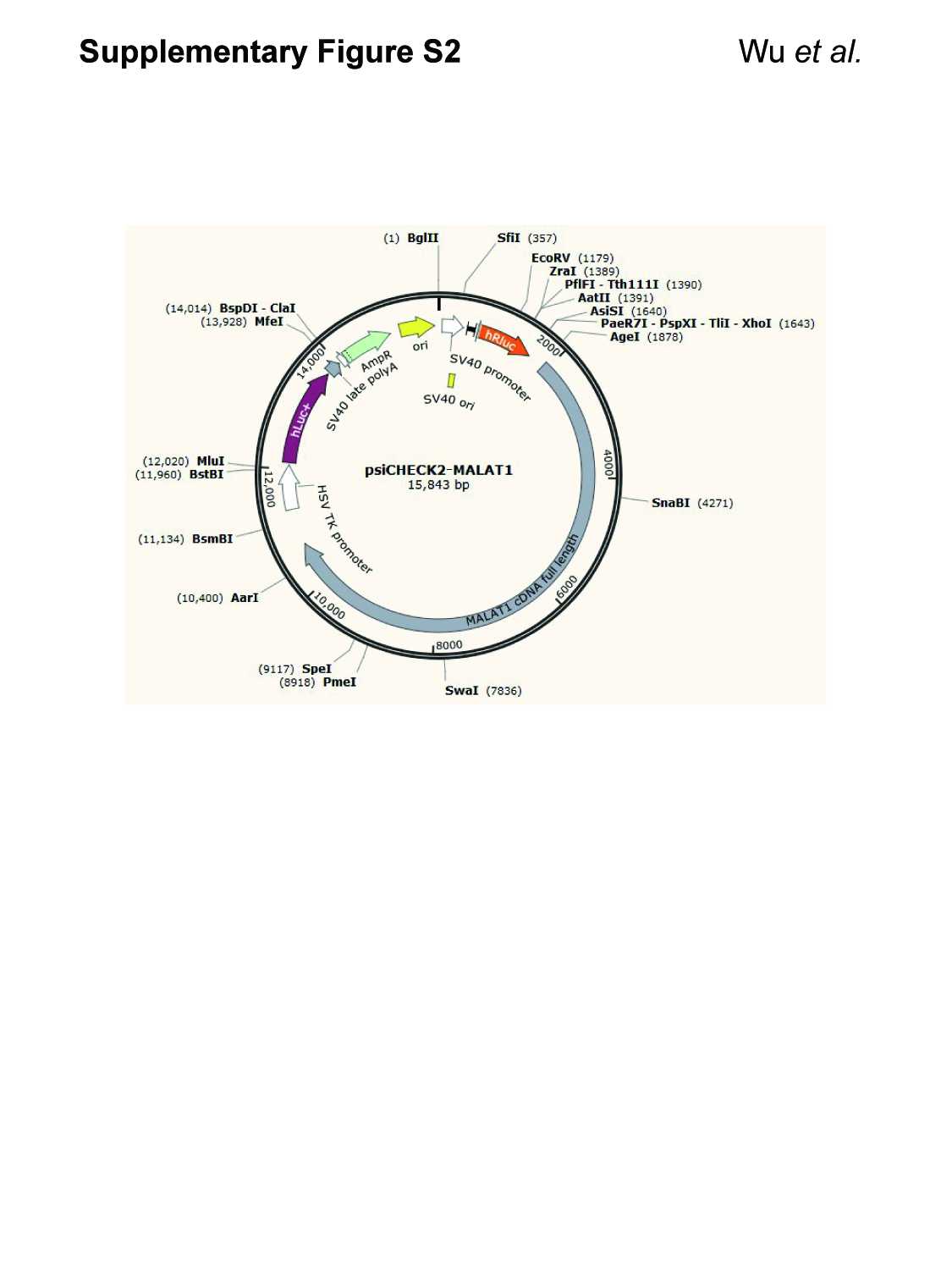

Supplement: Supplementary file 3 — Supplementary Figure S2 [file 41419_2020_2508_MOESM3_ESM.tif]

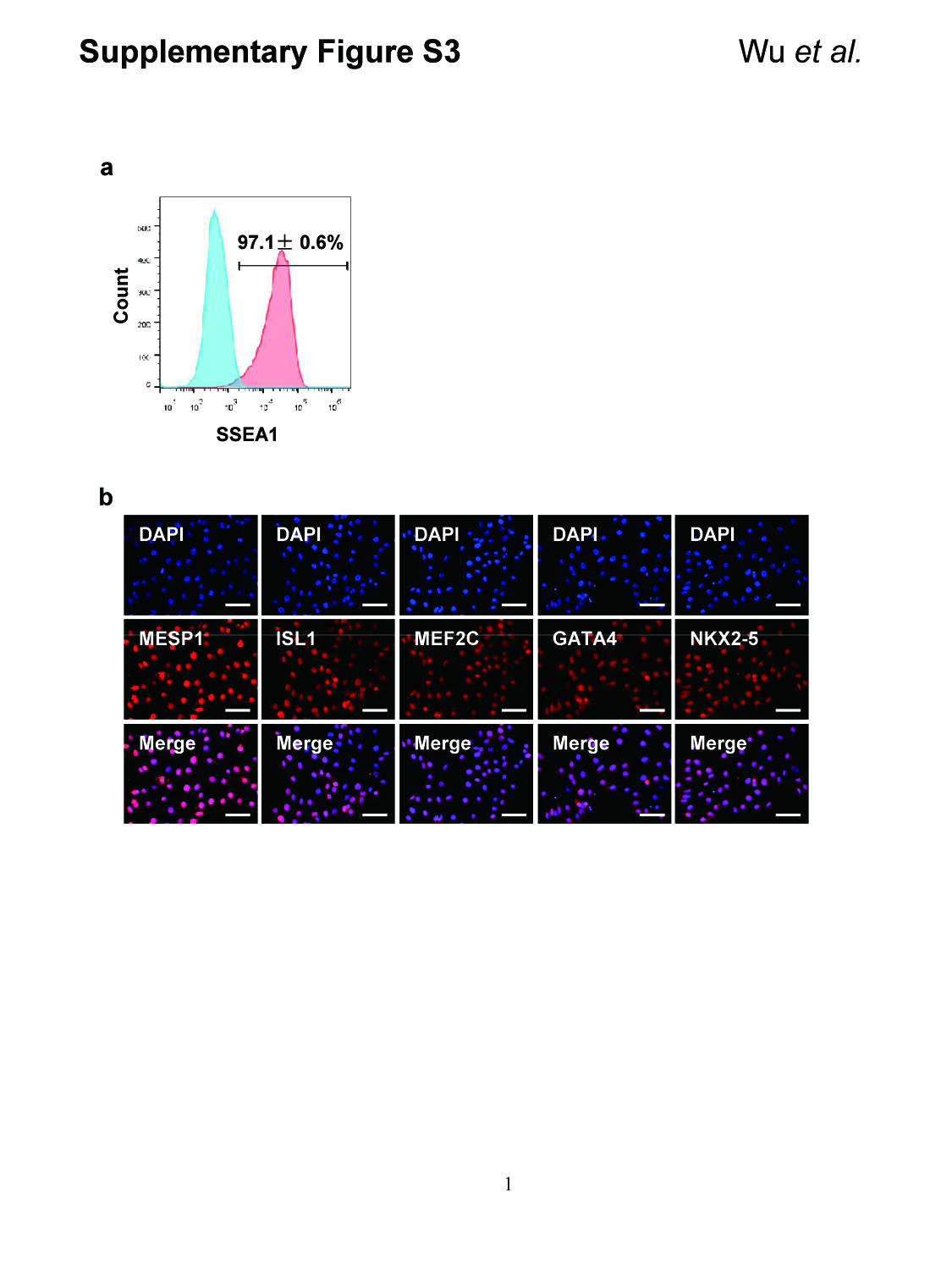

Supplement: Supplementary file 4 — Supplementary Figure S3 [file 41419_2020_2508_MOESM4_ESM.tif]

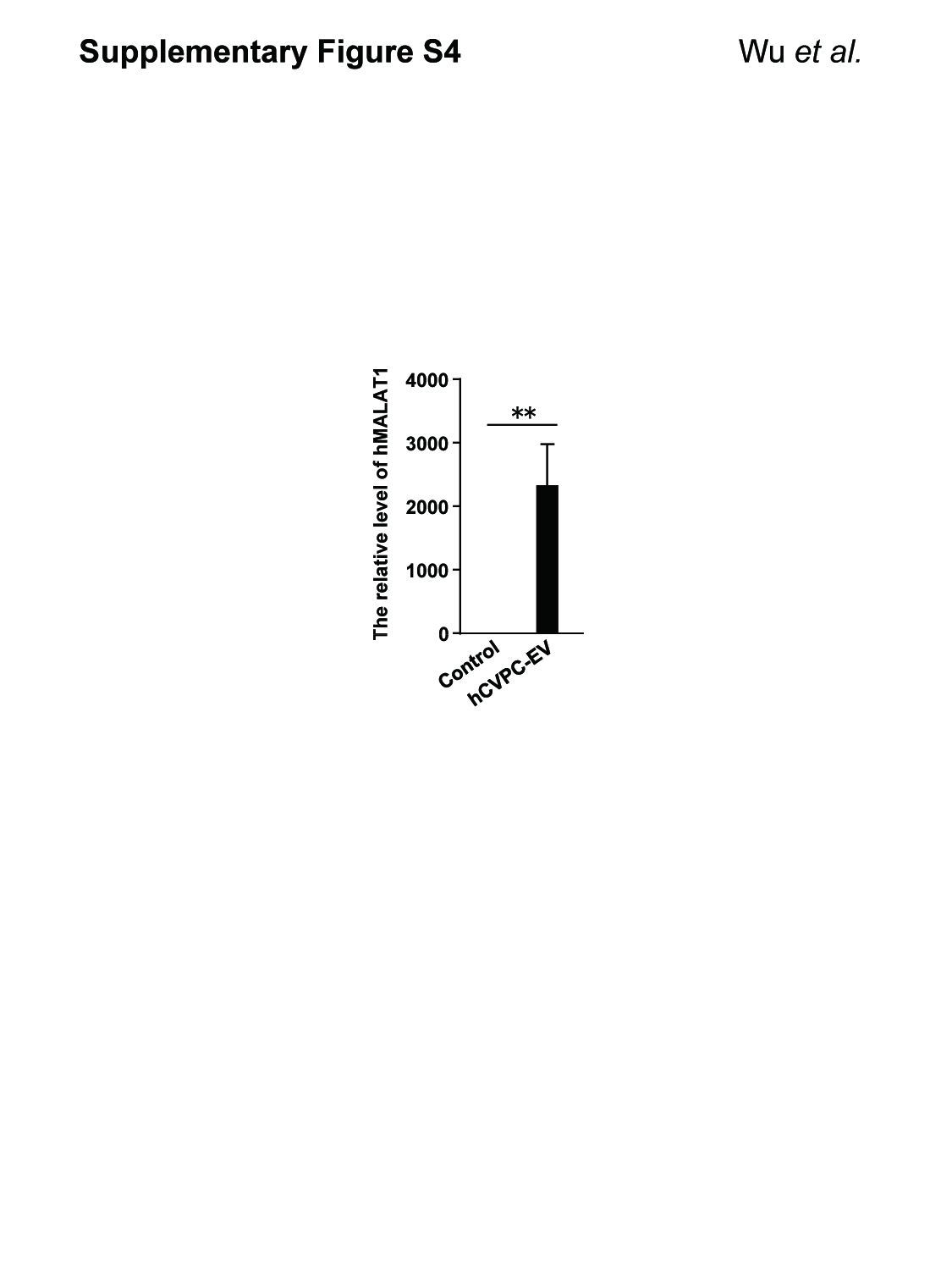

Supplement: Supplementary file 5 — Supplementary Figure S4 [file 41419_2020_2508_MOESM5_ESM.tif]

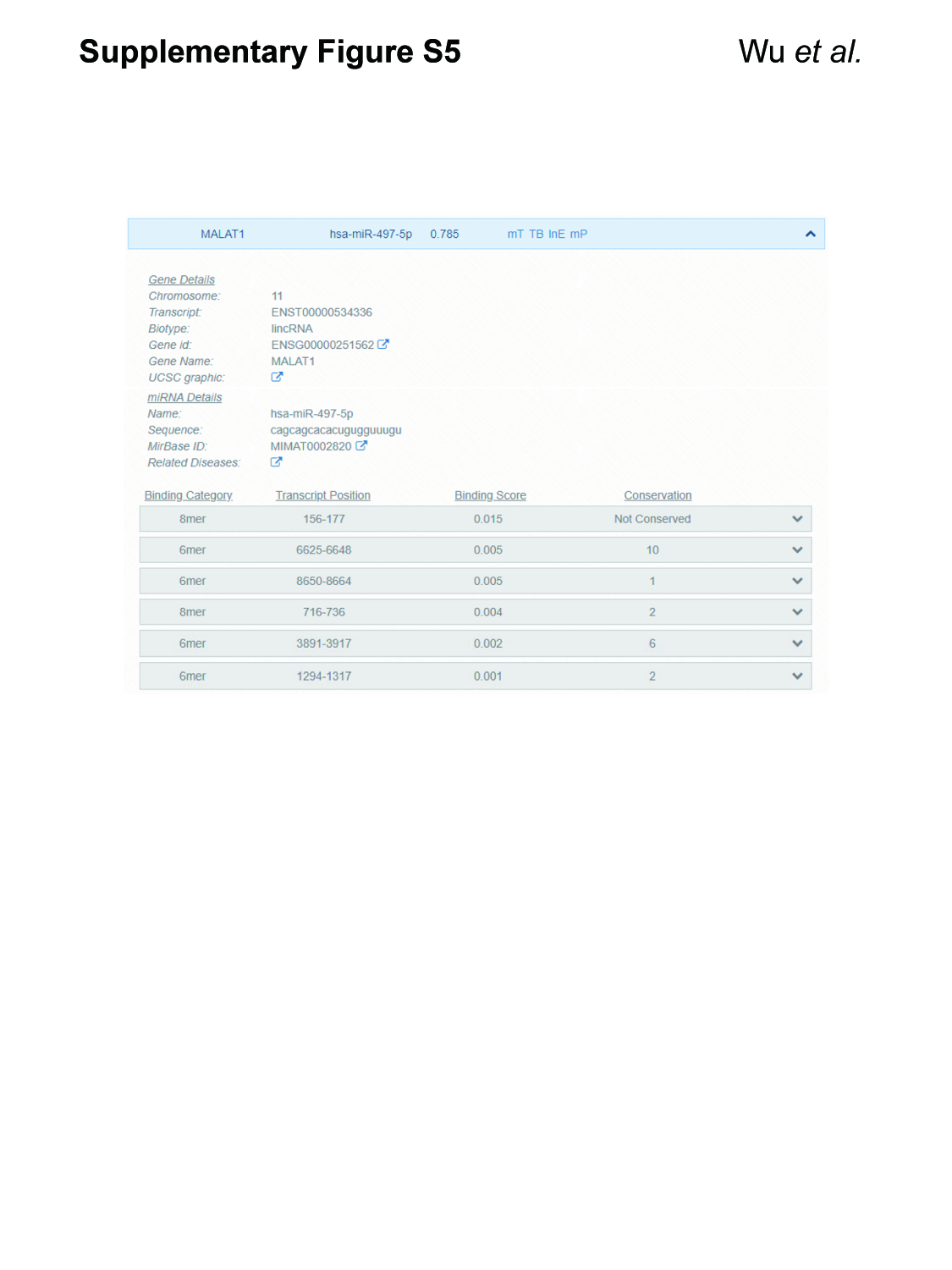

Supplement: Supplementary file 6 — Supplementary Figure S5 [file 41419_2020_2508_MOESM6_ESM.tif]

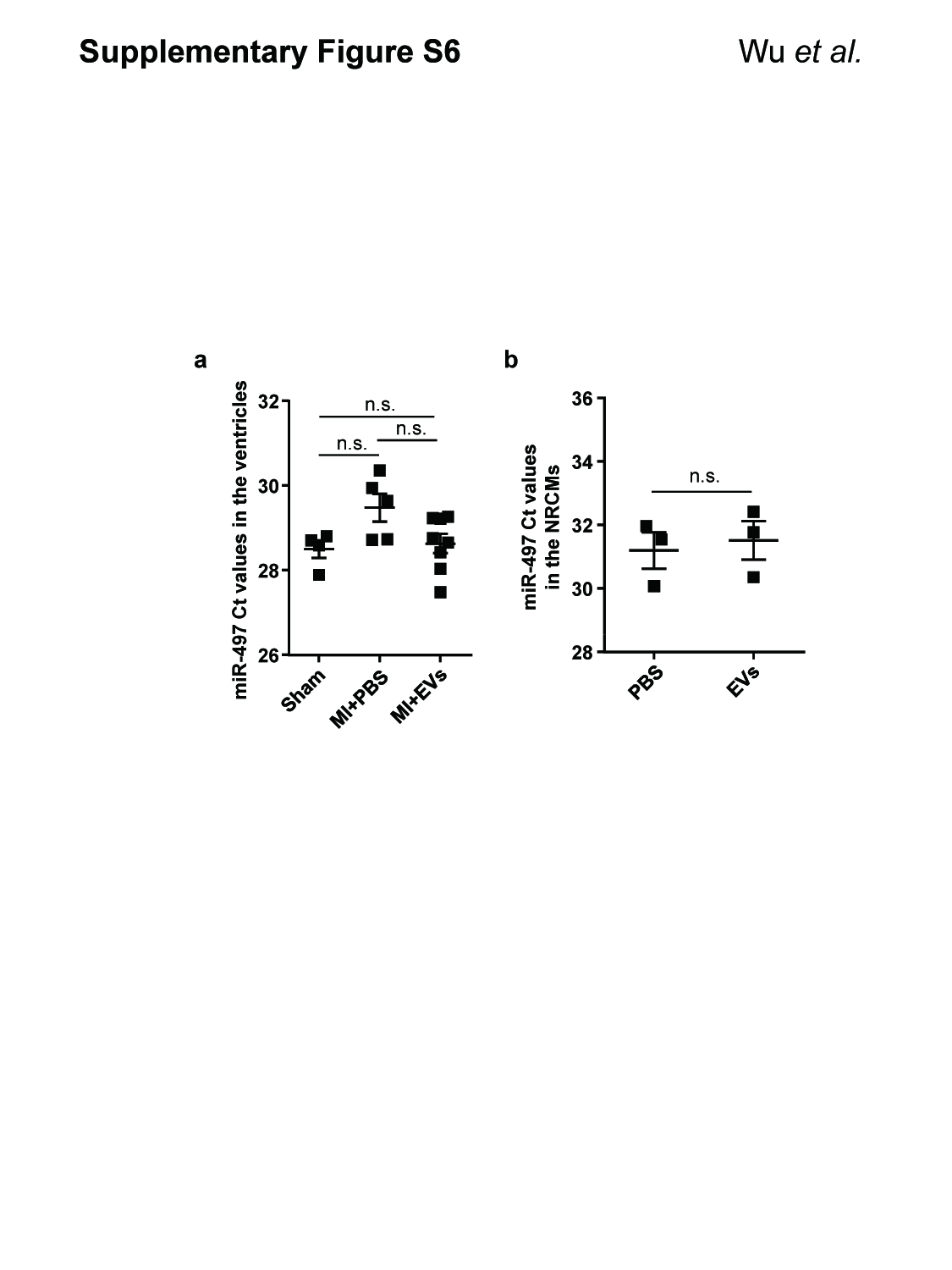

Supplement: Supplementary file 7 — Supplementary Figure S6 [file 41419_2020_2508_MOESM7_ESM.tif]
